# Lesions of the Pelvic Floor

**Published:** 1888-05

**Authors:** 


DR. HADRA'S BOOK.—*Lesions of the Vagina and Pelvic Floor*, with special reference to Uterine and Vaginal Prolapse. By B. E. Hadra, M. D., Austin, Texas; with 38 illustrations, Philadelphia. Records, McMullen & Co., limited, 1888, pages 326, cloth boards, price $2.00. Address Medical Register, 1519 Walnut street, Philadelphia, Pa.

This book was written about two years ago, and whatever omissions there may have been, or other defects, are corrected in an appendix. However, in a second edition much improvement can be made, and the work rendered more uniform. Still, as it is, it is the most comprehensive, yet condensed, review of the subject from a modern standpoint, extant. It is practical, and so arranged that the reader can at once find what he wishes without tedious search.

The question as to whether Alexander's or Olshausen's operation should be preferred, and performed for otherwise intractable cases of retroflexions, prolapse, etc., the author does not decide; opinion is divided on the subject, and a decision in favor of the one or the other would be premature; it yet remains to be seen which is the better operation for the specified purpose. These operations, however, are fully described by the author and their merits freely discussed and sufficient hints are given to assist the surgeon in a choice.

As to Colporrhaphy and Perineorrhaphy we are pleased to see that the author estimates their usefulness at its real worth; there is certainly too much refinement in devices for their performance, and he would simplify it. There is much that is new and original in the book, and we commend it to our readers as a real treat in that line.

The work is presented in good style ; it is printed on good paper in clear type, and altogether is a creditable production. We are much gratified to see the author testifies his appreciation of the many excellent qualities of head and heart,—of the *real worth* of our mutual and distinguished friend, Dr. T. J. Tyner, by dedicating this, his maiden effort, to him.

